# Guild-Level Microbiome Signature Associated with COVID-19 Severity and Prognosis

**DOI:** 10.1128/mbio.03519-22

**Published:** 2023-02-06

**Authors:** Mingquan Guo, Guojun Wu, Yun Tan, Yan Li, Xin Jin, Weiqiang Qi, Xiaokui Guo, Chenhong Zhang, Zhaoqin Zhu, Liping Zhao

**Affiliations:** a Department of Laboratory Medicine, Shanghai Public Health Clinical Center, Fudan University, Shanghai, China; b State Key Laboratory of Microbial Metabolism and Ministry of Education Key Laboratory of Systems Biomedicine, School of Life Sciences and Biotechnology, Shanghai Jiao Tong University, Shanghai, China; c Department of Biochemistry and Microbiology, School of Environmental and Biological Sciences and Center for Microbiome, Nutrition, and Health, New Jersey Institute for Food, Nutrition, and Health, Rutgers, The State University of New Jersey, New Brunswick, New Jersey, USA; d Rutgers-Jiaotong Joint Laboratory for Microbiome and Human Health, New Brunswick, New Jersey, USA; e Shanghai Institute of Hematology, State Key Laboratory of Medical Genomics, National Research Center for Translational Medicine at Shanghai, Ruijin Hospital, Shanghai, China; f School of Global Health, Chinese Center for Tropical Diseases Research, Shanghai Jiao Tong University School of Medicine, Shanghai, China; University of Michigan-Ann Arbor

**Keywords:** COVID-19, guild, gut microbiome

## Abstract

Coronavirus disease 2019 (COVID-19) severity has been associated with alterations of the gut microbiota. However, the relationship between gut microbiome alterations and COVID-19 prognosis remains elusive. Here, we performed a genome-resolved metagenomic analysis on fecal samples from 300 in-hospital COVID-19 patients, collected at the time of admission. Among the 2,568 high quality metagenome-assembled genomes (HQMAGs), redundancy analysis identified 33 HQMAGs which showed differential distribution among mild, moderate, and severe/critical severity groups. Co-abundance network analysis determined that the 33 HQMAGs were organized as two competing guilds. Guild 1 harbored more genes for short-chain fatty acid biosynthesis, and fewer genes for virulence and antibiotic resistance, compared with Guild 2. Based on average abundance difference between the two guilds, the guild-level microbiome index (GMI) classified patients from different severity groups (average AUROC [area under the receiver operating curve] = 0.83). Moreover, age-adjusted partial Spearman’s correlation showed that GMIs at admission were correlated with 8 clinical parameters, which are predictors for COVID-19 prognosis, on day 7 in hospital. In addition, GMI at admission was associated with death/discharge outcome of the critical patients. We further validated that GMI was able to consistently classify patients with different COVID-19 symptom severities in different countries and differentiated COVID-19 patients from healthy subjects and pneumonia controls in four independent data sets. Thus, this genome-based guild-level signature may facilitate early identification of hospitalized COVID-19 patients with high risk of more severe outcomes at time of admission.

## INTRODUCTION

Coronavirus disease 2019 (COVID-19), caused by the novel severe acute respiratory syndrome coronavirus 2 (SARS-CoV-2), has been a worldwide pandemic with a heavy toll on human health and the economy. Over 576 million people have been infected by SARS-CoV-2, with over 6 million deaths globally (1). Angiotensin-converting enzyme 2 (ACE-2), which is distributed in multiple tissues and widely expressed on the luminal surface of the gut, has been identified as a vital entry receptor of SARS-CoV-2 for promoting viral infection and replication (2). This can impair gut barrier and induce inflammation, which may disrupt the gut microbiome, contributing to cytokine storm and sepsis in already compromised patients with COVID-19 (2).

Recent studies have shown that dysbiosis of the gut microbiome and its related metabolites is closely associated with COVID-19 disease. These studies reveal the overall difference in the gut microbial composition between COVID-19 patients and healthy controls (3–11), association of microbial taxa and metagenomic functions with disease severity (3, 8, 9, 11) and persistent dysbiosis of the gut microbiota after recovery (3). The enrichment of pathobionts and depletion of beneficial microbes have been reported to be related to disease severity in COVID-19 (4, 7). However, these studies have suffered from small sample sizes and lack of cross-study validation and have missed microbiome signatures at admission for COVID-19 prognosis in hospitalized patients (4, 8–11). In addition, the reported findings are constrained due to analyzing the microbiome at low-resolution levels, such as species, genus, or even phylum, or broad metagenomic functional categories (3–11). In the gut microbial ecosystem, the strains/genomes are the minimum responding units to environmental perturbations and their response and contributions to the host are not constrained by taxonomy, even in the same species (12).

In this study, we obtained high-quality metagenome-assembled genomes (HQMAGs), which had completeness > 95%, contamination < 5%, and strain heterogeneity = 0, from metagenomically sequenced fecal samples collected from 300 in-hospital COVID-19 patients with mild, moderate, severe, and critical disease severities at the time of admission. We identified a guild-level microbiome signature of 33 HQMAGs. This signature classified patients with different severities, associating them with clinical parameters related to prognosis after 1 week in hospital and the death/discharge outcomes of critical patients. The capacity of this signature for classifying COVID-19 patients with different levels of severity and differentiating COVID-19 patients from pneumonia control and healthy individuals was validated in four independent data sets.

## RESULTS

### Overall structural changes of the gut microbiome were associated with disease severity in COVID-19 patients at admission.

From May to September 2020, we collected 330 stool samples from 300 in-hospital patients with COVID-19 confirmed by positive SARS-CoV2-2 reverse transcription-quantitative PCR (RT-qPCR) result. Among the 330 samples, 297 were collected from 297 patients at admission and 33 were collected from 29 patients during their hospitalization (Table S1 at https://github.com/nightkid03/SHCOVID-19). To profile the gut microbiome, metagenomic sequencing was performed on all 330 stool samples. To achieve strain/subspecies-level resolution, we reconstructed 2,568 nonredundant HQMAGs (two HQMAGs were collapsed into one if the average nucleotide identity [ANI] between them was > 99%) from the metagenomic data set. The HQMAGs accounted for more than 77.17% ± 0.23% (mean ± standard error of the mean [SEM]) of the total reads and were used as the basic variables for the subsequent microbiome analysis.

The 296 patients with a metagenomic data set at admission (one sample was discarded due to low mapping rate of the reads against HQMAGs) were classified into the (*n* = 88), moderate (*n* = 196), severe (*n* = 5), and critical (*n* = 7) groups based on their symptoms. Due to the limited sample sizes for severe and critical patients, we combined these two groups into one for the following analysis. There were significant differences in age between the patients with mild, moderate, and severe/critical symptoms (Kruskal-Wallis test, *P = *1.6 × 10^−14^); i.e., the more severe symptoms the patients had, the older they were (Fig. S1). There was no difference in gender among the 3 groups (chi-square test, *P = *0.22).

10.1128/mbio.03519-22.1 FIG S1 COVID-19 patients with more severe symptoms were older. Data points which do not share common compact letters are significantly different from each other (*P* < 0.05). Boxes show the medians and the interquartile ranges (IQRs); whiskers denote the lowest and highest values that lie within 1.5× the IQR from the first and third quartiles, outliers are shown as individual points. Kruskal-Wallis test followed by Dunn’s *post hoc* test (two-sided) was applied to compare the groups. Compact letters reflect the significant of the post hoc test (*P* < 0.05 as significant). Mild, *n* = 88; moderate, *n* = 196; severe/critical, *n* = 12. Download FIG S1, EPS file, 1.3 MB. Copyright © 2023 Guo et al. 2023 Guo et al. https://creativecommons.org/licenses/by/4.0/ This content is distributed under the terms of the Creative Commons Attribution 4.0 International license.

**FIG 1:**
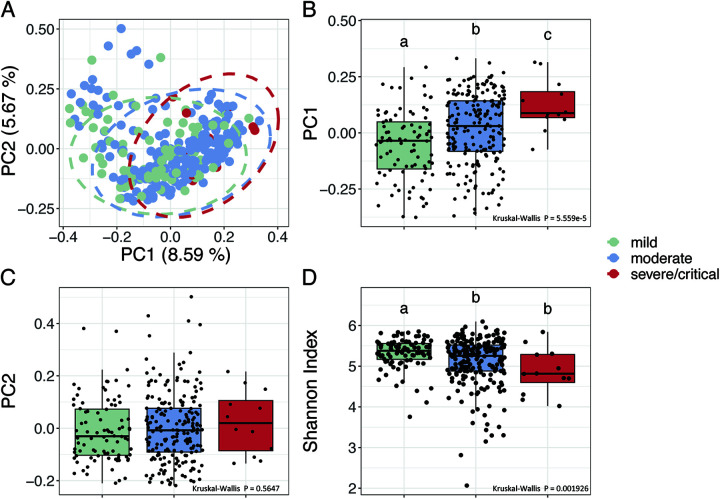
The overall structural variations of gut microbiota at admission are associated with disease severity in hospitalized COVID-19 patients. (A) Principal coordinate analysis (PCoA) based on Bray-Curtis calculated from abundance of the 2,568 genomes. (B) and (C) Comparison of the PC1 and PC2. (D) Comparison of alpha-diversity as indicated by Shannon index. Boxes show medians and interquartile ranges (IQRs); whiskers denote the lowest and highest values that were within 1.5× the IQR from the first and third quartiles, outliers are shown as individual points. Kruskal-Wallis test followed by Dunn’s *post hoc* test (two-sided) was used to compare groups. Compact letters indicate the significance of the *post hoc* test (*P* < 0.05 is significant). Mild, *n* = 88; moderate, *n* = 196; severe/critical, *n* = 12.

At admission, in the context of beta-diversity based on Bray-Curtis distance, principal coordinate analysis (PCoA) revealed separations of the gut microbiota along PC1, which was in accordance with the severity of symptoms (Fig. 1A to C). A single-factor permutational multivariate analysis of variance (PERMANOVA) test showed that both age (R^2^ = 0.0059, *P = *0006) and disease severity (R^2^ = 0.014, *P = *0.001) were significantly associated with the overall gut microbial composition. However, a marginal PERMANOVA test showed that when controlling for age, disease severity was still significantly associated with the overall gut microbial composition (R^2^ = 0.012, *P = *0.0002), but age was insignificant (R^2^ = 0.0033, *P = *0.5) when controlling for disease severity. This showed that in our data set, part of the variation in gut microbiota was uniquely associated with disease severity, which was independent of age. Pairwise comparisons between the 3 different severity groups via PERMANOVA showed that the gut microbial composition of the patients was significantly different from each other (mild versus moderate: R^2^ = 0.0081, *P = *0.0001; mild versus severe/critical: R^2^ = 0.026, *P = *0.0001; moderate versus severe/critical: R^2^ = 0.0079, *P = *0.0099). The distance between the mild and moderate groups was significantly smaller than that between the mild and the severe/critical groups (Fig. S2), which showed that the gut microbiota of severe/critical group was more different from the mild group compared with the moderate group. In regards to alpha-diversity, the Shannon index was highest in the mild group, followed by the moderate group and lowest in the severe/critical group (Fig. 1D, mild versus moderate: *P = *0.0046, mild versus severe/critical: *P = *0.0046, moderate versus severe/critical: *P = *0.086), which showed a continuous reduction in gut microbial diversity with increasing symptom severity. These results showed that the overall gut microbial structure was associated with symptom severity in COVID-19 patients.

10.1128/mbio.03519-22.2 FIG S2 Between-group Bray-Curtis distance. Boxes show the medians and IQRs; whiskers denote the lowest and highest values that lie within 1.5× the IQR from the first and third quartiles, outliers are shown as individual points. Kruskal-Wallis test followed by Dunn’s *post hoc* test (two-sided) was applied to compare the groups. Compact letters reflect the significant of the *post hoc* test (*P* < 0.05 is significant). Download FIG S2, TIF file, 7.1 MB. Copyright © 2023 Guo et al. 2023 Guo et al. https://creativecommons.org/licenses/by/4.0/ This content is distributed under the terms of the Creative Commons Attribution 4.0 International license.

### Two competing guilds were associated with disease severity of hospitalized COVID-19 patients at admission.

**FIG 2:**
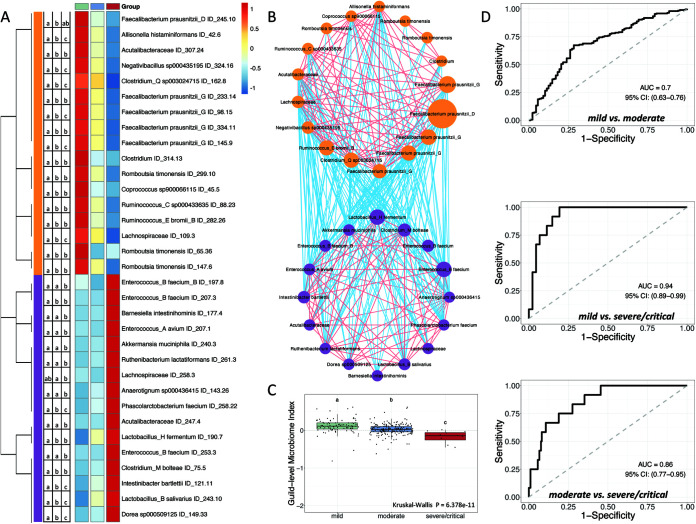
Two competing guilds composed of differentially abundant gut microbial genomes are associated with symptom severity in COVID-19 patients. (A) Heatmap of 33 high-quality metagenome-assembled genomes (HQMAGs) identified by redundancy analysis (RDA) and showing differences between the 3 severity groups. RDA analysis was conducted based on the Hellinger transformed abundance of all HQMAGs and used three symptom severity groups as environmental variables. HQMAGs with at least 5% of the variability in their abundance explained by constrained axes were selected. A Kruskal-Wallis test followed by Dunn’s *post hoc* test (two-sided) was used to test the differences between the 3 severity groups. Compact letters indicate the significance of the *post hoc* test (*P* < 0.05 is significant). Heatmap shows the mean abundance of each HQMAGs in each group. Abundance was scaled across each row. (B) Co-abundance network of the HQMAGs reflects two competing guilds. The co-abundance correlation between the HQMAGs were calculated using Fastspar (*n* = 296 subject). All significant correlations with Benjamini-Hochberg (BH)-adjusted *P* < 0.05 were included. Edges between nodes represent correlations. Red and blue colors indicate positive and negative correlations, respectively. Node size indicates the average abundance of the HQMAGs in 296 samples. Genomes were clustered into two guilds based on co-abundance correlation and complete linkage followed by weighted correlation network analysis (WGCNA) analysis. Node color indicates guild: Guild 1, orange; Guild 2, purple. (C) Comparison of guild-level microbiome index (GMI). Data points which do not share common compact letters are significantly different from each other (*P* < 0.05). Boxes show medians and IQRs; whiskers denote the lowest and highest values that lie within 1.5× the IQR from the first and third quartiles, outliers are shown as individual points. A Kruskal-Wallis test followed by Dunn’s *post hoc* test (two-sided) was applied to compare the groups. Compact letters indicate the significance of the test (*P* < 0.05). Mild, *n* = 88; moderate, *n* = 196; severe/critical, *n* = 12. (D) GMI supports classification of different COVID-19 symptom severities. AUROC (area under the receiver operating characteristic curve) is shown.

Specific HQMAGs that were associated with the COVID-19 symptom severity were identified by redundancy analysis (RDA) (Fig. S3). Out of the 2,568 HQMAGs, we found that 48 had at least 5% of their variability explained by the constraining variable, i.e., the three severity groups. Among the 48 HQMAGs, 17 were significantly higher in the mild group compared to the moderate and severe/critical groups, and these showed a continuous decrease alongside the symptom severity. These 17 HQMAGs included 5 from Faecalibacterium prausnitzii, 3 from Romboutsia timonensis, 2 each from *Ruminococcus* and *Clostridium*, and 1 each from Acutalibacteraceae, Allisonella histaminiformans, *Coprococcus*, Lachnospiraceae, and *Negativibacillus* (Fig. 2A). The abundance of 31 out of the 48 HQMAGs identified by RDA was higher in the severe/critical group compared with the mild and the moderate groups. Among these 31 HQMAGs, 16 showed significant differences between the three groups. These 16 HQMAGs included 4 from *Enterococcus*, 2 from *Lactobacillus*, and 1 each from Acutalibacteraceae, Akkermansia muciniphila, *Anaerotignum*, Barnesiella intestinihominis, Clostridium bolteae, *Dore*, Intestinibacter bartlettii, Lachnospiraceae, Phascolarctobacterium faecium, and Ruthenibacterium lactatiformans (Fig. 2A). We then focused on the 17 mild group-enriched HQMAGs and 16 severe/critical group-enriched HQMAGs because they were all identified by RDA analysis and were significantly different between the 3 severity groups.

10.1128/mbio.03519-22.3 FIG S3 Tri-plot of redundancy analysis (RDA) of the microbial composition based on the 2,568 high quality metagenome-assembled genomes (HQMAGs). The three symptom severity groups were used as environmental variables. Samples are indicated by dots. HQMAGs with at least 5% of the variability in their abundance explained by RDA1 and RDA2 are indicated by blue arrows. RDA analysis was conducted based on the Hellinger transformed abundance. Download FIG S3, EPS file, 1.4 MB. Copyright © 2023 Guo et al. 2023 Guo et al. https://creativecommons.org/licenses/by/4.0/ This content is distributed under the terms of the Creative Commons Attribution 4.0 International license.

Because bacteria in the gut ecosystem are not independent but rather form coherent functional groups (aka “guilds”) to interact with each other and affect host health (13), we applied co-abundance analysis to these 33 HQMAGs to explore the interactions between them and find potential guild structures with hierarchical clustering and weighted correlation network analysis (WGCNA) (14). Interestingly, the 33 HQMAGs organized themselves into two guilds. The 17 HQMAGs with significantly higher abundance in mild group were positively interconnected with each other and formed Guild 1. The 16 severe/critical group-enriched HQMAGs were positively correlated with each other as Guild 2. Meanwhile, there were only negative correlations between the two guilds, suggesting a potentially competitive relationship between them (Fig. 2B).

To explore the genetic basis underlying the associations between the two guilds and symptom severities, we performed a genome-centric analysis of the metagenomes of the two competing guilds. A previous study showed that a lack of short-chain fatty acids (SCFAs) is significantly correlated with disease severity in COVID-19 patients (7). For the terminal genes for the butyrate biosynthetic pathways (i.e., *but*, *buk*, *atoA/D*, and *4Hbt*) (15), 7 HQMAGs in Guild 1 harbored the *but* gene, while only 1 HQMAGs in Guild 2 possessed this gene (Fisher’s exact test, *P = *0.039) (Fig. S4). Four HQMAGs in Guild 1 harbored the *buk* gene, while no HQMAGs in Guild 2 had this gene (Fisher’s exact test, *P = *0.10). The other butyrate biosynthetic terminal genes were not found in the HQMAGs in either guild. The numbers of HQMAGs encoding genes for acetate and propionate production were similar in the two guilds (Fig. S4). From a pathogenicity perspective, both guilds had 12 HQMAGs encoding virulence factor (VF) genes. However, Guild 1 had 17 VF genes from 3 VF categories, while Guild 2 had 58 VF genes from 5 VF categories (Fig. S5A). In terms of antibiotic resistance genes (ARGs), 3 genomes in Guild 1 encoded 10 ARGs and 5 genomes in Guild 2 encoded 14 ARGs (Fig. S5B). Taken together, these data showed that the two competing guilds had different genetic capacities, with Guild 1 being more beneficial and Guild 2 more detrimental. Thus, the genetic difference between the two guilds may help explain their associations with disease severity in COVID-19 patients.

10.1128/mbio.03519-22.4 FIG S4 Differences in genetic capacity of short-chain fatty acid (SCFA) biosynthesis between the two guilds. Heatmap shows the gene copy numbers of But, butyryl-coenzyme A (butyryl-CoA); acetate CoA transferase; Buk, butyrate kinase; FTHFS, formate-tetrahydrofolate ligase for acetate production; ScpC, propionyl-CoA succinate-CoA transferase; and Pct, propionate-CoA transferase for propionate production. Download FIG S4, TIF file, 4.4 MB. Copyright © 2023 Guo et al. 2023 Guo et al. https://creativecommons.org/licenses/by/4.0/ This content is distributed under the terms of the Creative Commons Attribution 4.0 International license.

10.1128/mbio.03519-22.5 FIG S5 Differences in genetic capacity for pathogenicity and antibiotic resistance between the two guilds. (A) Bar plot shows the number of genes encoding virulence factors (VF) and classes of VFs. (B) Bar plot shows the number of antimicrobial resistance genes (ARGs) and the corresponding antibiotic resistance types. Download FIG S5, EPS file, 0.6 MB. Copyright © 2023 Guo et al. 2023 Guo et al. https://creativecommons.org/licenses/by/4.0/ This content is distributed under the terms of the Creative Commons Attribution 4.0 International license.

**FIG 3:**
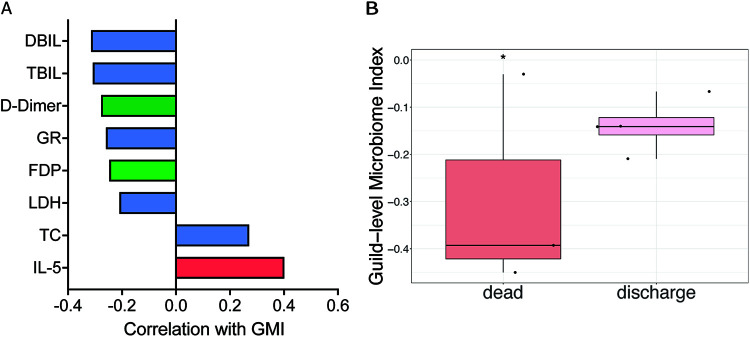
The two competing guilds at admission are associated with COVID-19 severity in hospitalized patients on day 7 after admission and with endpoint in critical patients. (A) Bar plot shows the correlations between the GMI at admission and clinical parameters of COVID-19 in hospitalized patients on day 7. Age-adjusted partial Spearman’s correlation was calculated. Correlations with BH-adjusted *P* < 0.1 are shown. Blue bar, biochemical indicators; green bar, coagulation indicators; red bar, immune indicators. (B) GMI at admission associated with death/discharge outcomes of critical COVID-19 patients. A two-sided Mann-Whitney test was used to determine significance. *, *P* < 0.05. Death, *n* = 3; discharge, *n* = 4.

We then calculated the guild-level microbiome index (GMI) based on the average abundance difference between guilds 1 and 2 to reflect the dominance of Guild 1 over Guild 2. At admission, the GMI was highest in the mild group, followed by the moderate group, and was lowest in the severe/critical group (Fig. 3C; mild versus moderate: *P = *2.46 × 10^−7^; mild versus severe/critical: *P = *6.57 × 10^−9^; moderate versus severe/critical: *P = *1.59 × 10^−4^). The GMI reached an AUROC (area under the receiver operating characteristic curve) of 0.7 and an AUPRC (area under the precision-recall curve) of 0.8, with a baseline of 0.69 to differentiate between the mild and moderate groups; an AUROC of 0.94 and AUPRC of 0.59 with a baseline of 0.12 to differentiate between the mild and severe/critical groups; and an AUROC of 0.86 and AUPRC of 0.32 with a baseline of 0.06 to differentiate between the moderate and severe/critical groups (Fig. 3D and Fig. S6A–C). This result indicates the feasibility of using the GMI as a biomarker to differentiate between different symptom severity groups of COVID-19 patients.

10.1128/mbio.03519-22.6 FIG S6 Guild-level microbiome index (GMI) supports classification according to different COVID-19 symptom severities and differentiation of COVID-19 subjects from pneumonia control and healthy individuals, assessed by AUPRC (area under the precision-recall curve). Download FIG S6, TIF file, 4.3 MB. Copyright © 2023 Guo et al. 2023 Guo et al. https://creativecommons.org/licenses/by/4.0/ This content is distributed under the terms of the Creative Commons Attribution 4.0 International license.

### Gut microbiome signature was associated with the COVID-19 prognosis of hospitalized patients.

To explore whether our microbiome signature at admission is associated with the prognosis of COVID-19 patients during hospitalization, we calculated correlations between GMI at admission and 72 different clinical parameters on day 7 of hospitalization. Two and six clinical parameters on day 7 showed significantly (partial Spearman’s correlation; Benjamini-Hochberg [BH]-adjusted *P < *0.1) positive and negative correlations, respectively, with the GMI values at admission after adjusting for age (Fig. 3A). Regarding immune indicators, interleukin (IL)-5 is secreted chiefly by Th2 cells and is essentially anti-inflammatory but also involved in several allergic responses (16). Some studies have revealed higher levels of IL-5 in severe cases than in mild cases (17, 18). However, others have shown that IL-5 levels have no correlations with COVID-19 and showed no differences between different severity groups (19, 20). Here, we found positive correlations between the GMI at admission and IL-5 levels after 1 week. The effects of the microbiome on particular cytokines and its subsequent influences on COVID-19 require further study. Coagulation disorder occurred during the early stage of COVID-19 infection (21). D-dimer and fibrin degradation product (FDP) levels increased in COVID-19 patients and were correlated with clinical classification (21, 22). Moreover, elevated D-dimer and FDP levels are significant indicators of severe COVID-19 and poor prognosis (21–24). Here, a higher GMI at admission was correlated with lower D-dimer and FDP levels after 1 week. Regarding biochemical indicators, compared with health subjects, total cholesterol (TC) was significantly lower in COVID-19 patients and decreased with increasing severity (25, 26). A meta-analysis showed that a reduction in TC was significantly associated with increased mortality in COVID-19 patients, and TC may assist with early risk stratification (26). Hypocalcemia has been reported to be common in COVID-19 patients (27). Higher total bilirubin (TBIL) was associated with a significant increase in the severity of COVID-19 infection (28). Moreover, COVID-19 patients with an elevated TBIL at admission had a higher mortality rate (28). In addition to TBIL, increased direct bilirubin (DBIL) has been reported as an independent indicator of complications and mortality in COVID-19 patients (29). In particular, DBIL levels on day 7 of hospitalization are advantageous for predicting the prognosis of COVID-19 in severe/critical patients (29). Lactate dehydrogenase (LDH) has been associated with worse outcomes in viral infection. One meta-analysis showed that LDH could be used as a COVID-19 severity marker and a predictor of survival (30). Here, the GMI at admission was positively correlated with TC and negatively correlated with TBIL, DBIL, and LDH after 1 week. These results suggest that the gut microbiome signature in early stages of the disease may reflect the clinical outcomes of COVID-19 in hospitalized patients.

Moreover, in our cohort, 3 patients died, all of which were in the critical group at admission. Compared with the other 4 discharged critical patients, the 3 dead patients were significantly younger (Fig. S7). The GMIs of the 3 dead patients at admission were significantly lower than those of the 4 discharged critical patients (Fig. 3B). This suggests an association between the microbiome signature and the final outcome in critical hospitalized COVID-19 patients. Although interesting, this result should be interpreted with caution given the small sample size.

10.1128/mbio.03519-22.7 FIG S7 In patients who had critical COVID-19 at admission, those who died were significantly younger than those who were discharged. Mann-Whitney test (two-sided) was used. Dead, *n* = 3; discharged, *n* = 4. Download FIG S7, EPS file, 1.2 MB. Copyright © 2023 Guo et al. 2023 Guo et al. https://creativecommons.org/licenses/by/4.0/ This content is distributed under the terms of the Creative Commons Attribution 4.0 International license.

### The microbiome signature was validated in independent studies.

**FIG 4:**
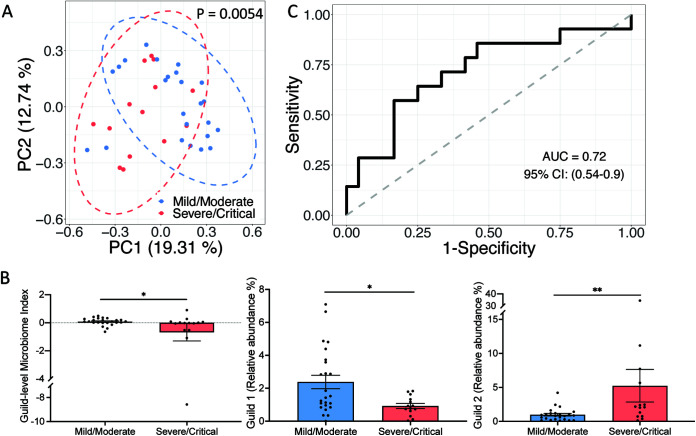
Genome-based microbiome signature enables classification of COVID-19 patients from different severity groups in an independent Chinese cohort. (A) PCoA based on Bray-Curtis distance calculated from the abundance of the 33 HQMAGs. Permutational multivariate analysis of variance (PERMANOVA) test showed significant differences in the composition of the 33 HQMAGs between the two groups. (B) Significant differences in GMI and abundances of guilds 1 and 2 between mild/moderate and severe/critical COVID-19 patients. Bar plot summarizes the mean and standard error of the mean (SEM). Mann-Whitney test (two-sided) was used to compare groups. Mild/moderate, *n* = 24; severe/critical, *n* = 14. **, *P* < 0.01; *, *P* < 0.05. (C) GMI supports classification according to different COVID-19 symptom severities.

We then asked whether this genome-based microbiome signature would be applicable in other COVID-19 cohorts. To answer this question, we used the genomes of the 33 HQMAGs as references to perform read-recruitment analysis, a common method for estimating the abundances of reference genomes from metagenomes (31, 32). In an independent study, which included 24 mild/moderate and 14 severe/critical COVID-19 patients from China (9), we validated the associations between the microbiome signature and different COVID-19 severities. In this validation data set, the two patient groups had even distributions of age, gender, and comorbidities, preventing potential biases for our validation. On average, the 33 HQMAGs accounted for 4.39% ± 0.90% (mean ± SEM) of the total abundance of the gut microbial community. In the context of beta-diversity, as measured via Bray-Curtis distance, the composition of the microbiome signature significantly differed between mild/moderate and severe/critical COVID-19 patients (Fig. 4A). GMI and abundance of Guild 1 were significantly higher in the mild/moderate patients, while the abundance of Guild 2 was significantly higher in the severe/critical patients (Fig. 4B). Moreover, GMI had a discriminatory power of AUROC = 0.72 and AUPRC = 0.63 with a baseline of 0.37 to differentiate the two severity groups (Fig. 4C and Fig. S6D).

**FIG 5:**
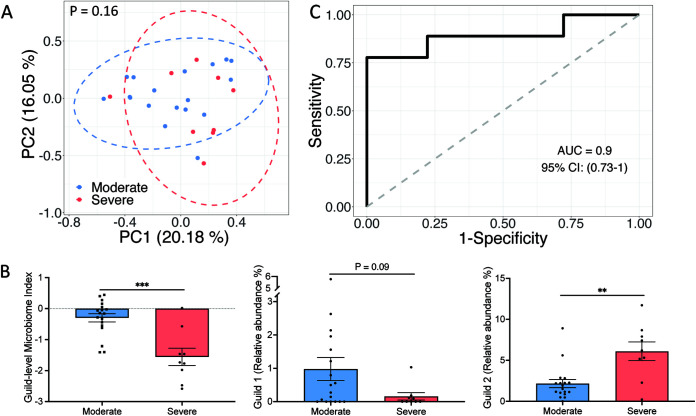
Genome-based microbiome signature enables classification of COVID-19 patients from different severity groups in an independent American cohort. (A) PCoA based on Bray-Curtis distance calculated from the abundance of the 33 HQMAGs. PERMANOVA test showed significant differences in the composition of the 33 HQMAGs between the two groups. (B) Significant differences in GMI and abundances of guilds 1 and 2 between moderate and severe COVID-19 patients. Bar plot summarizes mean and SEM. Mann-Whitney test (two-sided) was applied to compare groups. Moderate, *n* = 18; severe, *n* = 9. ***, *P* < 0.001; **, *P* < 0.01. (C) GMI supports classification according to different COVID-19 symptom severities.

To further test the applicability of the microbiome signature in different geographies, we included metagenomic sequencing data from 18 moderate and 9 severe COVID-19 patients from the United States (33) and applied the same validation process. On average, the 33 HQMAGs accounted for 4.18% ± 0.59% (mean ± SEM) of the total abundance of the gut microbial community. Although the composition of the microbiome signature between the moderate and severe COVID-19 patients was not significantly different based on Bray-Curtis distance (Fig. 5A), the GMI and abundance of Guild 1 were significantly higher in the moderate patients, while the abundance of Guild 2 was significantly higher in the severe patients (Fig. 5B). GMI had a discriminatory power of AUROC = 0.9 and AUPRC = 0.89 with a baseline of 0.33 to differentiate the two severity groups (Fig. 5C and Fig. S6E). These results validated our findings of the associations between genome-resolved microbiome signature and COVID-19 disease severity in different geographies.

**FIG 6:**
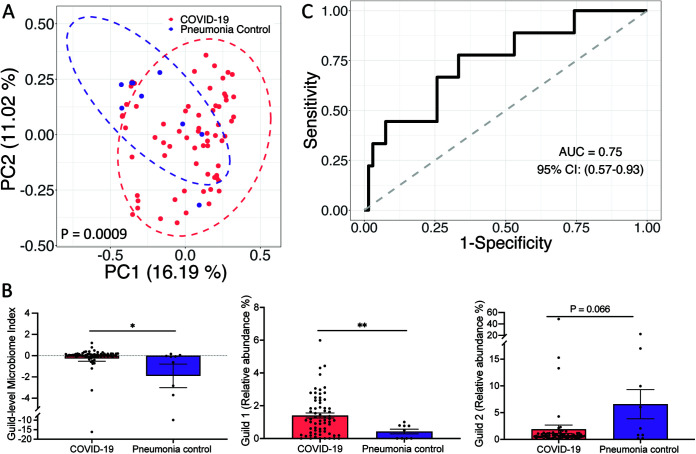
Genome-based microbiome signature enabled distinction between COVID-19 and pneumonia subjects in an independent data set. (A) PCoA based on Bray-Curtis distance calculated from the abundance of the 33 HQMAGs. (B) Significant differences in GMI and abundances of guilds 1 and 2 between COVID-19 and pneumonia subjects. Bar plot summarizes the mean and SEM. Mann-Whitney test (two-sided) was applied to compare groups. COVID-19, *n* = 66; pneumonia, *n* = 9. **, *P* < 0.01; *, *P* < 0.05. (C) GMI supports classification between COVID-19 and pneumonia control group.

Because this microbiome signature was associated with COVID-19 disease and was able to classify COVID-19 severity, we were interested in determining whether it could classify COVID-19 and non-COVID-19 controls as well. We first included metagenomic sequencing data from 66 COVID-19 patients (first sample after admission), of which 47 were mild/moderate, and 9 community-acquired pneumonia controls which were negative for COVID-19^7^. The genomes of the 33 HQMAGs were used as reference genomes to perform read-recruitment analysis. On average, the 33 HQMAGs accounted for 3.75% ± 0.74% (mean ± SEM) of the total abundance of the gut microbial community. In the context of beta-diversity as measured via Bray-Curtis distance, the composition of the microbiome signature between the two groups was significantly different (Fig. 6A). The GMI and abundance of Guild 1 were significantly higher in the COVID-19 group, while abundance of Guild 2 was higher in the pneumonia control group (Fig. 6B). GMI had a discriminatory power of AUROC = 0.75 and AUPRC = 0.32 with a baseline of 0.12 to differentiate the two groups (Fig. 6C and Fig. S6F).

**FIG 7:**
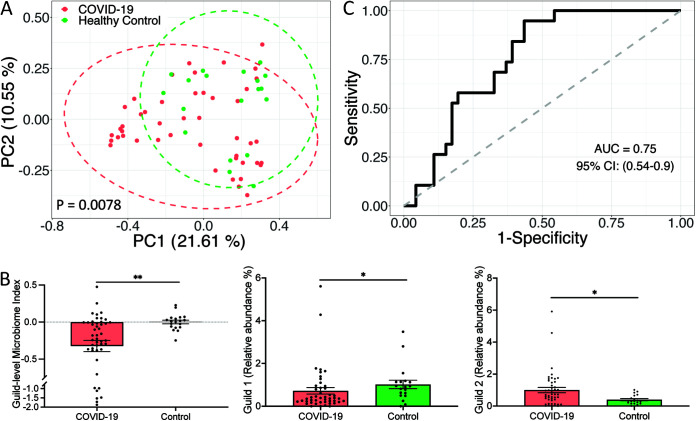
Genome-based microbiome signature enables distinction between COVID-19 subjects and heathy controls in an independent data set. (A) PCoA based on Bray-Curtis distance calculated from the abundance of the 33 MAGs. PERMANOVA test showed significant differences in the composition of the 33 MAGs between the two groups. (B) Significant differences in GMI and abundances of guilds 1 and 2 between COVID-19 and healthy subjects. Bar plot summarizes the mean and SEM. Mann-Whitney test (two-sided) was applied to compare groups. COVID-19, *n* = 46; healthy control, *n* = 19. *, *P* < 0.05; **, *P* < 0.01. (C) GMI supports classification of COVID-19 and healthy control subjects.

Next, we included metagenomic sequencing data from 46 COVID-19 patients and 19 age- and sex-matched healthy controls from the study conducted by Li et al. (34). On average, the 33 HQMAGs accounted for 1.61% ± 0.12% (mean ± SEM) of the total abundance of the gut microbial community. Based on the Bray-Curtis distance, the PCoA plot revealed a separation between the COVID-19 patients and healthy subjects (Fig. 7A). Compared with the healthy controls, COVID-19 patients had a significantly lower GMI and abundance of Guild 1 but a higher abundance of Guild 2 (Fig. 7B). These results suggest that SARS-CoV-2 infection is associated with altered composition of the 33 HQMAGs. GMI had a discriminatory power of AUROC = 0.75 and AUPRC = 0.9 with a baseline of 0.71 to differentiate the COVID-19 patients and healthy controls (Fig. 7C and Fig. S6G). These showed that the microbiome signature was related to host health and could be used as a biomarker to differentiate the COVID-19 subjects from the pneumonia controls and healthy subjects.

## DISCUSSION

In the current study, a genome-based microbiome signature, composed of 33 HQMAGs at the time of admission, was found to be associated with the severity and prognosis of COVID-19 in hospitalized patients. With these 33 genomes as a reference, we were also able to validate the microbiome signature in data sets collected from four independent studies.

We arrived at this finding by way of a unique analytical strategy for the microbiome data set. Previous studies relied on reference databases to profile gut microbial composition at taxonomic levels and explored the relationships between different taxa and COVID-19^3–11^. Our strategy used a reference-free discovery approach which does not need any prior knowledge. This allowed us to keep the novel part of the data set intact. In addition, the use of high-quality draft genomes in our study ensured the highest possible resolution for identifying microbiome signatures associated with COVID-19, overcoming the pitfalls of taxon-based analysis (13). In previous studies based on taxon-level analysis, Enterococcus faecium, Enterococcus avium, and Akkermansia muciniphila have been reported to be enriched in severe/critical COVID-19 patients and positively correlated with symptom severity (3, 9). In our results, a total of 28 *A. muciniphila*, 2 *E. avium*, and 5 E. faecium HQMAGs were assembled in our data set, but only 3 strains of E. faecium and 1 each of *A. muciniphila* and *E. avium* were enriched in the severe/critical group, suggesting that not all strains from these 3 species were associated with COVID-19 severity. Another example is that Faecalibacterium prausnitzii, a key producer of SCFAs, is consistently depleted in COVID-19 patients and negatively correlated with disease severity (3, 4); however, in our results, only half of the *F. prausnitzii* HQMAGs in our data set were negatively associated with COVID-19 symptom severity. These results indicate that the associations between gut microbiota and COVID-19 are strain/genome-specific. This means that even species-level analysis may not provide the necessary resolution to reveal associations of gut microbiome with COVID-19.

In addition to identifying COVID-19 associated gut microbiota at the genome level, we used guild-based analysis to reveal potential interactions among key gut bacteria via a co-abundance network. We found that the genomes enriched in the mild/moderate group and the genomes enriched in the severe/critical group formed two guilds, Guild 1 and Guild 2, respectively. The genomes in Guild 1 had higher SCFA-producing genetic capacity, while the Guild 2 genomes contained more VF- and ARG-encoding genes. Reduced abundance of SCFA-producing pathways has been correlated with more adverse clinical outcomes in COVID-19 patients (7). The expression levels of VF and ARG, as measured by metatranscriptomic sequencing, were significantly higher in COVID-19 patients compared with the healthy and non-COVID-19 pneumonia controls (35). Higher abundance of Guild 1 and lower abundance of Guild 2 were associated with reduced severity in our COVID-19 patients. Such a two-competing-guilds structure, in which one beneficial guild and one detrimental guild compete with each other and influence host health, has been reported as a core microbiome signature associated with various chronic diseases (36). Our findings suggest that such two competing guild microbiome signatures may also be applicable to infectious diseases.

In our study cohort, GMI based on the average abundance difference between the two guilds was able to discriminate between different symptomatic severity groups of COVID-19 patients at admission. This capacity of GMI to discriminate COVID-19 symptom severities has been further validated in independent cohorts from China and United States (9, 33). Moreover, GMI had the capacity to distinguish COVID-19 subjects from pneumonia controls and healthy subjects in two other independent studies (7, 34). These indicate the feasibility of using this microbiome signature risk stratification for COVID-19 patients. It is worth validating the applicability of the microbiome signature in COVID-19 diagnosis in cohorts across additional ethnicities and geographies.

A recent mouse-model study showed that SARS-COV-2 infection alone caused gut microbiome dysbiosis and gut epithelial cell alterations, with an increased number of goblet cells and a decreased number of Paneth cells (37). This dysbiotic gut microbiome may play a role in modulating host immune responses and outcomes of COVID-19 patients by translocating potential pathogens or their antigens into systemic circulation (37) and decreasing production of metabolites such as SCFAs and l-isoleucine (7). In the current study, we found that the microbiome signature, which was related to COVID-19 severity at admission, was associated with COVID-19 prognosis. The two competing guilds’ microbiome signatures at admission were associated with several coagulation, hemogram, and biochemical indicators of hospitalized patients after 1 week. These indicators included D-dimer, FDP, TC, TBIL, DBIL, and LDH, which have been reported to play essential roles in the host response to COVID-19 infection and disease progression (21–30). The microbiome signature may serve as an early predictor of COVID-19 prognosis because it was positively associated with bio-clinical parameters that have inverse relationships with poor prognosis and negatively associated with those that have direct relationships with poor prognosis. More importantly, early time-point variations of the two competing guilds’ microbiome signatures were correlated with later changes in these prognosis related bio-clinical parameters. These results suggest that dysbiosis of the gut microbiota may play a pivotal role in triggering more severe symptoms after patients are infected with SARS-CoV-2. More mechanistic studies, such as time-series experiments involving transplanting gut microbiota from COVID-19 patients with different disease severities or transplanting different combinations of the isolates from the two competing guilds into germ-free mice with or without SARS-COV-2 infection, and more gut microbiota-targeted intervention studies on COVID-19 patients, are needed to further understand the relationships between gut microbiota and COVID-19 prognoses.

Early identification and treatment of high-risk patients is critical for improving COVID-19 prognosis when the end of the pandemic is not yet in sight due to emerging SARS-CoV-2 variants such as Omicron. Screening hospitalized COVID-19 patients at the time of admission using our genome-based guild-level microbiome signature may facilitate early identification of patients at high risk of more severe outcomes so they can be put under intensive surveillance and preventive care.

## MATERIALS AND METHODS

### Ethics statement.

This study procedure was reviewed and approved by the Ethics Committee of Shanghai Public Health Clinical Center (SHPHC, no. YJ-2020-S080-02), and informed written consent were obtained from all subjects according to the Declaration of Helsinki. All experimental procedures were performed in strict accordance with the biosafety operation guidelines of the SARS-CoV-2 Laboratories of the National Health and Family Planning Commission (no. 2020 [70]) and the Shanghai Municipal Health and Family Planning Commission (no. 2020 [8]). Table S1 at https://github.com/nightkid03/SHCOVID-19 lists sample collection and disease severity information.

### Subject recruitment and sample collection.

This study was retrospectively conducted at the Shanghai Public Health Clinical Center, a designated hospital for COVID-19 treatment in East China. In total, 337 COVID-19 patients were recruited for this study; all patients were typed and grouped based on clinical symptoms by senior clinicians in strict accordance with the criteria of the Diagnosis and Treatment Plan for SARS-CoV-2 (trial version 7) issued by the General Office of the National Health Commission. The clinical data of the study subjects, including patient epidemiology (age, gender, disease classification, length of hospital stay, duration of disease, clinical outcomes) and respective clinical laboratory test results (hematologic, clinical chemistry, coagulation, immune inflammatory indices, and radiographic indications) were stored in a computerized database in the hospital medical record system. Stool samples were collected within 48 h of admission from all patients from May to September 2020, ensuring that all patients did not receive antiviral, antibiotic, probiotic, hormone, or other drug interventions. About 100 mg of each patient’s feces was collected in a stool collection tube and frozen immediately at −80°C until processing.

### Clinical laboratory examination and data collection.

All laboratory tests were conducted at the department of laboratory medicine in the Shanghai Public Health Clinical Center. A Sysmex XN-1000 automated hematology analyzer (Hisense Meikang Medical Electronics, Shanghai Co., Ltd.) and its supporting test reagents were used to analyze blood routine tests, including white blood cell count, lymphocyte count, platelet count, %neutrophils, %monocytes, %lymphocytes, hemoglobin, hypersensitive C-reactive protein, etc. Biochemical parameters such as albumin, amylase, cholinesterase, lactate, lactate dehydrogenase, alkaline phosphatase, glucose, creatinine, uric acid, and prealbumin were measured by a biochemical immunoassay workstation (ARCHITECT 3600J, Abbott Laboratories Co., USA). Urine routine (pH value, specific gravity, urobilinogen, leukocyte esterase, nitrite, urine protein, glucose, ketone body, bilirubin, and occult blood) was measured by a Cobas 6500 urine dry chemical analysis system and supporting test strips (Roche, Switzerland). For the coagulation indicators, an STA Compact Max was used to measure fibrinogen, D-dimer, fibrinogen degradation products, prothrombin time, activated partial thromboplastin time, thrombin time, etc.

### Plasma cytokine measurements.

A FACS Canto II Flow cytometer (BD Biosciences, USA) was used for lymphocyte analysis, CD3^+^ cell counts, CD4^+^ cell counts, CD8^+^ cell counts, CD19^+^ cell counts, CD16^+^ CD56^+^ cell counts, and to determine CD4^+^/CD8^+^ percentage. Plasma cytokine-related parameters, including IL-1β, IL-2, IL-4, IL-5, IL-6, IL-8, IL-10, IL-12P70, IL-17, tumor necrosis factor alpha, interferon alpha, and interferon gamma, were measured using a microsphere array kit and a FACS Canto II cytometer (Raisecare Biotechnology, China).

### Gut microbiome analysis.

**(i) DNA extraction and metagenomic sequencing.** The laboratory procedures in this section were performed by trained laboratory personnel under the condition of tertiary protection in a biosafety level 2 (BSL2)-qualified laboratory. DNA was extracted from fecal samples using the bead-beating method as previously described (38); a QIAamp PowerFecal Pro DNA kit (Qiagen, Germany) was used to perform DNA extraction according to the manufacturer’s instructions. Briefly, fecal samples (~100 mg) were dissolved by Powerlyzer lysate in a PowerBead Pro Tube, followed by vigorous shaking for 10 min and centrifugation. Total genomic DNA was captured on a silica membrane in a spin-column. DNA was then washed and eluted. An *A*_260_/*A*_280_ ratio of ~1.8, concentration, and curve observations were used to assess the quality of DNA extraction. Qualified DNA samples were ready for downstream application. Metagenomic sequencing was performed using an Illumina HiSeq 3000 at GENEWIZ Co. (Beijing, China). Cluster generation, template hybridization, isothermal amplification, linearization, and blocking, denaturing, and hybridization of the sequencing primers were performed according to the workflow specified by the service provider. Libraries were constructed with an insert size of approximately 500 bp followed by high-throughput sequencing to obtain paired-end reads with 150 bp in the forward and reverse directions. Table S2 at https://github.com/nightkid03/SHCOVID-19 shows the number of raw reads for each sample.

**(ii) Data quality control.** Trimmomatic (39) was used to trim low-quality bases from the 3′ end, remove low quality reads, and remove reads of <60 bp, with the parameters leading:6 trailing:6, slidingwindow:4:20 minlen:60. Reads that could be aligned to the human genome (H. sapiens, UCSC hg19) were removed (aligned with Bowtie2 [40] using -reorder -no-hd -no-contain -dovetail). Table S2 at https://github.com/nightkid03/SHCOVID-19 shows the number of high-quality reads of each sample for further analysis.

**(iii) *De novo* assembly, abundance calculation, and taxonomic assignment of genomes.** *De novo* assembly was performed for each sample using MEGAHIT (41) (-min-contig-len 500, -presets meta-large). The assembled contigs were further binned using MetaBAT 2 (42) and MaxBin 2 (43). A refinement step was then performed using the bin_refinement module from MetaWRAP (44) to combine and improve the results generated by the 2 binners. The quality of the bins was assessed using CheckM (45). Bins with completeness > 95%, contamination < 5%, and strain heterogeneity = 0 were retained as high-quality draft genomes (Table S3 at https://github.com/nightkid03/SHCOVID-19). The assembled high-quality draft genomes were further dereplicated by using dRep (46). DiTASiC (47), which applies kallisto for pseudo-alignment (48) and a generalized linear model for resolving shared reads among genomes, was used to calculate the abundance of the genomes in each sample, estimated counts with *P* > 0.05 were removed, and all samples were downsized to 30 million reads (one sample at admission with a read-mapping ratio of ~32%, which could not be well represented by the high-quality genomes, were removed in further analyses). Taxonomic assignment of the genomes was performed using GTDB-Tk (49) (Table S4 at https://github.com/nightkid03/SHCOVID-19).

**(iv) Gut microbiome functional analysis**. Prokka (50) was used to annotate genomes. KEGG Orthologue (KO) IDs were assigned to the predicted protein sequences in each genome by HMMSEARCH against KOfam using KofamKOALA (51). Antibiotic resistance genes were predicted using ResFinder (52) with the default parameters. Identification of virulence factors was based on the core set of the Virulence Factors of Pathogenic Bacteria Database (VFDB [53], downloaded July 2020). Predicted protein sequences were aligned to the reference sequence in VFDB using BLASTP (best hist with E value < 1e-5, identity > 80%, and query coverage > 70%). Genes encoding formate-tetrahydrofolate ligase, propionyl-CoA:succinate-CoA transferase, propionate CoA-transferase, 4Hbt, AtoA, AtoD, Buk, and But were identified as described previously (54).

**(v) Gut microbiome co-abundance network construction and analysis.** Fastspar (55), a rapid and scalable correlation estimation tool for microbiome study, was used to calculate the correlations between the genomes with 1,000 permutations at each time point based on the abundances of the genomes across all patients, and correlations with BH-adjusted *P* < 0.05 were retained for further analysis. The co-abundance network was visualized using Cytoscape v3.8.1 (56). Complete linkage based on the co-abundance correlations followed by WGCNA analysis (14) was used to identify the guilds.

**(vi) Definition of guild-level microbiome index.** We defined the GMI using the abundance of the 33 QHMAGs and their relationships. For each individual sample, the GMI of sample *j*, denoted as GMI_j_, was calculated as follows:

(1) $$I_{\text{j}}^{\text{guild1}}=\underset{i\in N}{\sum }A_{\text{ij}}$$

(2) $$I_{\text{j}}^{\text{guild2}}=\underset{i\in M}{\sum }A_{\text{ij}}$$

(3) $${\text{GMI}}_{\text{j}}=\frac{I_{\text{j}}^{\text{guild1}}}{\left|N\right|}-\frac{I_{\text{j}}^{\text{guild2}}}{\left|M\right|}\;$$

Where *A*_ij_ is the relative abundance of HQMAG *i* in sample *j*; *N* and *M* are subsets of HQMAGs in guilds 1 and 2, respectively; and |*N*| and |*M*| are the sizes of these two sets. GMI = 0 indicates equality between guilds 1 and 2. Theoretically, the range of GMI is −100 to 100.

**(vii) Validation in an independent cohort.** Metagenomic sequencing data from 24 mild/moderate and 14 severe/critical COVID-19 patients were downloaded from the European Nucleotide Archive (ENA) database under PRJNA792726 (9) (Table S5 at https://github.com/nightkid03/SHCOVID-19). The metagenomic sequencing data from 18 moderate and 9 were downloaded from ENA under PRJNA660883 (33) (Table S5). The metagenomic sequencing data from 66 COVID-19 patients (first sample after admission) and 9 community-acquired pneumonia controls that were negative for COVID-19 were downloaded from ENA under PRJNA689961 (7) (Table S5). The metagenomic sequencing data from 46 COVID-19 patients and 19 healthy controls were downloaded from ENA under PRJEB43555 (34) (Table S5). KneadData (https://huttenhower.sph.harvard.edu/kneaddata/) was applied to perform quality control of the raw reads with the following parameters: -decontaminate-pairs strict, -run-trim-repetitive, -bypass-trf, -trimmomatic-options = “slidingwindow:4:20 minlen:60.” Reads that could be aligned to the human genome were identified and removed in KneadData by aligning reads against the Homo sapiens hg37 genome. The abundance of the 33 MAGs were estimated by using Coverm v0.6.1 (https://github.com/wwood/CoverM) with the following parameters: coverm genome –min-read-aligned-percent = 90 -min-read-percent-identity = 99 -m relative_abundance.

### Statistical Analysis.

Statistical analysis was performed in R version 4.1.1. A Kruskal-Wallis test followed by Dunn’s *post hoc* test (two-sided) was used to compare the different severity groups. Redundancy analysis was conducted based on the Hellinger transformed abundance to find specific gut microbial members associated with COVID-19 severity. Both single-factor and marginal PERMANOVA tests including both age and symptom severity were used to compare overall gut microbial composition. AUROC and AUPRC were used to evaluate the capacity of GMI to discriminate between groups using the R packages pROC and PRROC (57), respectively. AUROC considers the trade-offs between sensitivity and specificity and compares the performance of classifiers with a baseline value of 0.5 for a random classifier. AUPRC, which considers the trade-offs between precision and recall with a baseline that equals the proportion of positive cases in all samples, was used as a complementary assessment, particularly for highly imbalanced data sets.

### Code availability.

Parameters of the bioinformatic tools applied in the study are listed in Materials and Methods. Scripts and command lines related to the current study can be found at https://github.com/nightkid03/SHCOVID-19.

### Ethics and inclusion statement.

We have carefully considered research contributions and authorship criteria when involved in multi-region collaborations involving local researchers to promote greater equity in research collaborations.

### Data availability.

The metagenomic sequencing data for the current study have been deposited into the CNGB Sequence Archive (CNGB) of the China National GenBank Database (CNGBdb) (58) under accession no. CNP0003849. Supplementary tables can be found at https://github.com/nightkid03/SHCOVID-19.
