# Erratum for Guo et al., “Guild-Level Microbiome Signature Associated with COVID-19 Severity and Prognosis”

**DOI:** 10.1128/mbio.00149-25

**Published:** 2025-01-29

**Authors:** Mingquan Guo, Guojun Wu, Yun Tan, Yan Li, Xin Jin, Weiqiang Qi, Xiaokui Guo, Chenhong Zhang, Zhaoqin Zhu, Liping Zhao

## ERRATUM

Volume 14, no. 1, e03519-22, 2023, https://doi.org/10.1128/mbio.03519-22. Page 14, Acknowledgments, first sentence: “(no. 8210061470)” should read “(no. 82101847).”

