# Supplementary figures and images for: Guild-Level Microbiome Signature Associated with COVID-19 Severity and Prognosis
Source: mBio. 2023 Feb 6;14(1):e03519-22. doi: 10.1128/mbio.03519-22 (PMC9973266; doi:10.1128/mbio.03519-22)

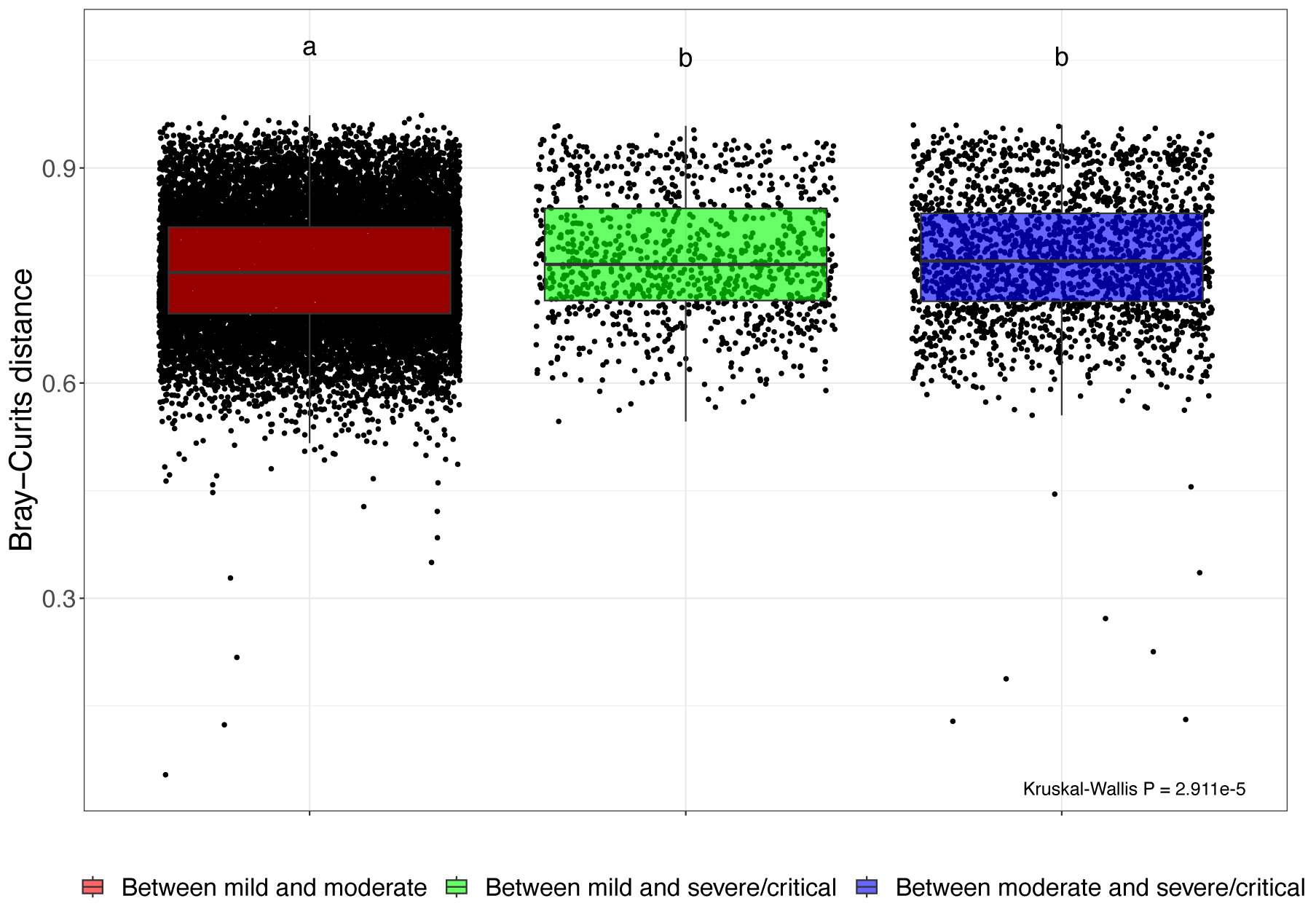

Supplement: FIG S2 [file mbio.03519-22-s0002.tif]

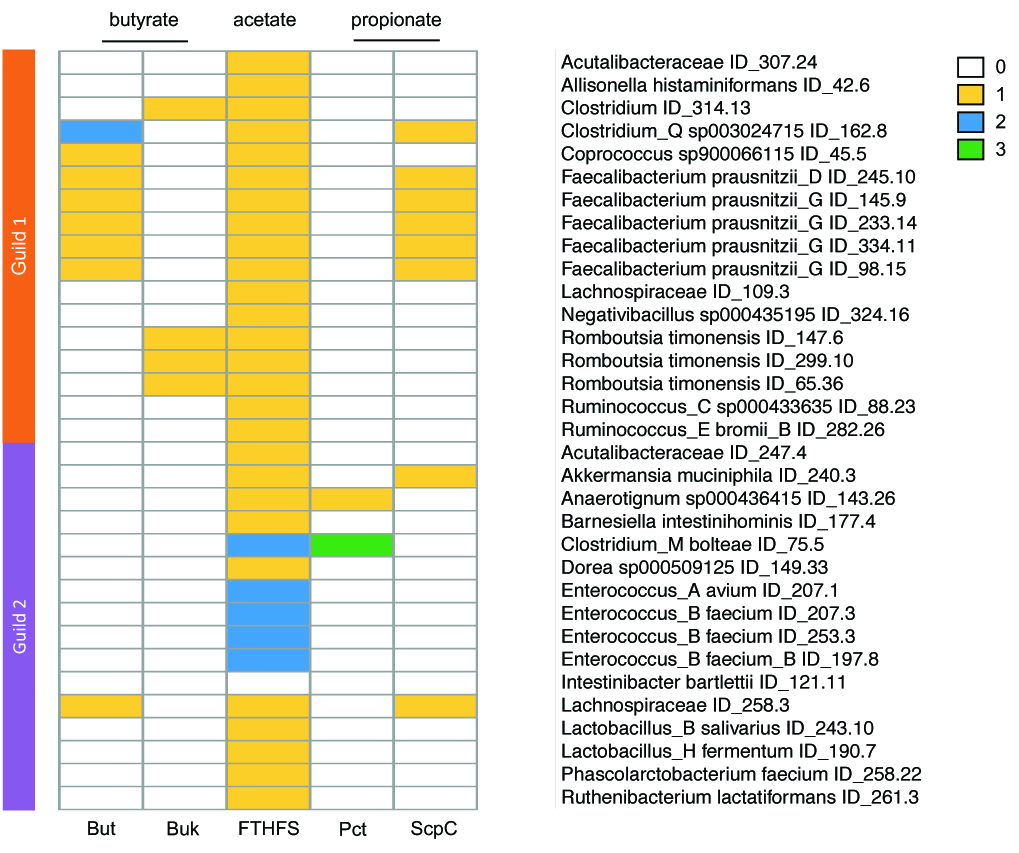

Supplement: FIG S4 [file mbio.03519-22-s0004.tif]

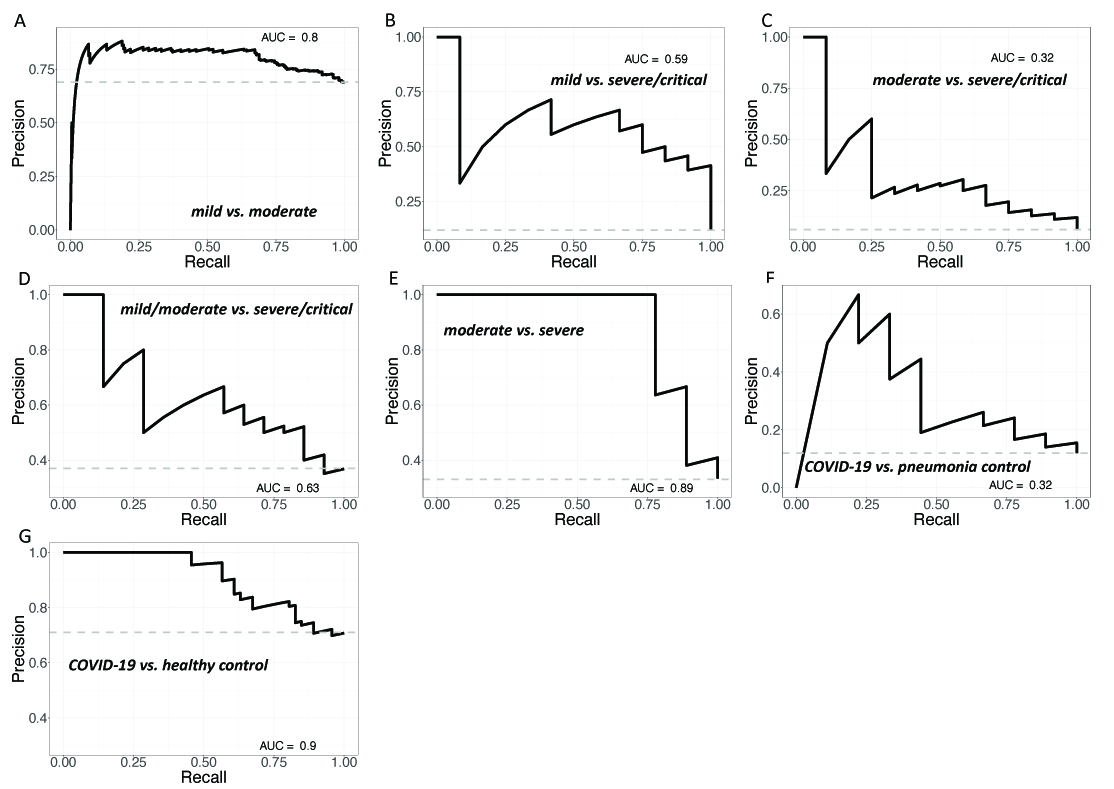

Supplement: FIG S6 [file mbio.03519-22-s0006.tif]
